# The Cyclopædia of Practical Medicine

**Published:** 1845-01

**Authors:** 


Art. VI.—*The Cyclopædia of Practical Medicine.* Edited by John Forbes, M.D., F.R.S., Alexander Tweedie, M.D., F.R.S., and John Conolly, M.D. Revised, with additions, by Robley Dunglison, M.D. Philadelphia. Lea & Blanchard. 1844.

Since our December number was issued we have received parts XVI., XVII., XVIII., and XIX., of this elaborate work, which is progressing with perfect regularity towards its completion.

At a season when pneumonia is one of our prevailing diseases, the following extract from the Cyclopædia will be read with interest:

"*Application of the Treatment.*—We have only to point out here the manner in which the several remedies which we have described may be combined in the treatment of individual cases. Where the case is at all severe, the treatment with tartar-emetic, or with calomel and opium, should be commenced immediately after the first bleeding, and continued uninterruptedly until an impression shall have been produced on the disease. It is not safe, however, with either of these remedies to lay aside blood-letting; if sensible relief does not ensue in the course of five or six hours after the first full bleeding, this measure must be repeated as before recommended. In the most acute and inflammatory cases, uncomplicated with gastric disease, we have sometimes combined the mercurial with the antimonial treatment, by giving a pill containing from six to ten grains of calomel, with from half a grain to a grain and a halt of opium every four or six hours, and the tartar-emetic draught before prescribed, in the middle of that interval; and where the tolerance is established soon, the effect of this treatment is very powerful. If the bowels are too much acted on, the hydrargyrum cum cretâ in double quantity may be substituted for the calomel. When an amelioration takes place in the symptoms, the mercury may be omitted, and the case left to the tartar-emetic, and whatever depletion or counter-irritation may be required. If the attack of pneumonia is very recent, and accompanied with a sharp stitch in the side, or catch in the breathing, a full dose of opium immediately after a large bleeding, as recommended by Dr. Armstrong, will sometimes be found sufficient to cut short the disease. This plan can only be adopted where the bleeding has been so copious as to produce a great and real impression on the heart's action, almost, if not quite, amounting to syncope. The dose of opium should be large; three or four grains of the aqueous extract, or if the pain and tendency to reaction are urgent, from 30 to 60 minims of the liquor opii sed., are the preparations to which we give the preference. Even in this case we think it advisable to give from six to twelve grains of calomel, or from ten to twenty grains of blue pill, with, or soon after, the opium; the mercury does not interfere with the sedative operation of the latter, and by preserving the balance of the secretions, it prevents those functional derangements which sometimes follow the use of opium even in this way. If the disease has in any degree passed into the second stage, and even if the first has lasted more than twenty-four hours, there can be little hope of stifling it with opium, and we must then resort to the alterative powers of tartar-emetic or mercury, with whatever blood-letting the case may bear. Blisters can seldom with advantage be resorted to, until all fulness and hardness of the pulse and heat of skin have subsided; and either of these symptoms, or even the continuance of a fixed pain, would counter-indicate the use of the decoction of senega, or any of the mild tonics which prove useful in the decline of the inflammation.

"If, from the copious expectoration of pus and the physical signs of a cavity in the lungs, it is probable that an abscess has been formed, it may be necessary to support the strength by a stronger tonic, as the sulphate of quinine or bark with a mineral acid; but the expediency of this must depend on a complete predominance of the symptoms of weakness over those of inflammation. In case of gangrene, some of the medicines of a supposed antiseptic power may be given. Drs. Graves and Stokes, in a case of this kind, gave the chloride of lime in the dose of three grains with one of opium three times a day, with the effect of removing the fetor from the expectoration, and for a time improving the symptioms.

We have seen the same medicine in solution apparently produce the same result; but we cannot expect much from the operation of such agents. The principal object in case of gangrene is to support the general strength, and counteract the noxious influence of the dead matter, until it can be thrown off from the system."

